# Micro–Macro Relationships in the Simulation of Wave Propagation Phenomenon Using the Discrete Element Method

**DOI:** 10.3390/ma12244241

**Published:** 2019-12-17

**Authors:** Jerzy Rojek, Nikhil Madan, Szymon Nosewicz

**Affiliations:** Institute of Fundamental Technological Research Polish Academy of Sciences, Pawińskiego 5B, 02-106 Warsaw, Poland; nmadan@ippt.pan.pl (N.M.); snosew@ippt.pan.pl (S.N.)

**Keywords:** discrete element method, wave propagation, elastic properties, micro–macro relationships

## Abstract

The present work is aimed to investigate the capability of the discrete element method (DEM) to model properly wave propagation in solid materials, with special focus on the determination of elastic properties through wave velocities. Reference micro–macro relationships for elastic constitutive parameters have been based on the kinematic hypothesis as well as obtained numerically by simulation of a quasistatic uniaxial compression test. The validity of these relationships in the dynamic analysis of the wave propagation has been checked. Propagation of the longitudinal and shear wave pulse in rectangular sample discretized with discs has been analysed. Wave propagation velocities obtained in the analysis have been used to determine elastic properties. Elastic properties obtained in the dynamic analysis have been compared with those determined by simulation of the quasistatic compression test.

## 1. Introduction

Wave propagation is a fundamental phenomenon which is encountered frequently in different natural processes, for instance, earthquakes, and engineering problems such as non-destructive testing or structures subjected to impact loading. A range of numerical schemes are used in literature to discretize the wave equation in space and investigate the wave propagation phenomenon, e.g., finite different schemes as elastodynamic finite integration technique [1,2] and local interaction simulation approach [3], finite element method [4], spectral element method [5], discontinuous Galerkin method [6], or boundary element method [7]. The discrete element method (DEM) is often used for the analysis of different problems of geomechanics involving wave propagation [8,9,10]. Owing to its simplistic mathematical framework based on Newtonian equations of motion, the DEM has emerged as a numerical tool of frequent choice for investigating the systems with inhomogeneities, or discontinuities existing in the material or appearing under loading. Although the main reason to use the DEM in such problems is not its capability to represent elastic waves, but this feature is important since wave propagation is an inherent phenomenon in any dynamic problem.

In the DEM, a material is represented by a large assembly of discrete elements interacting with each other by contact. Various contact laws representing physical effects such as elasticity, viscosity, damage and friction [11,12,13,14] can be used to define the inter-particle contact interactions. It is the cumulative behaviour of these micro level interactions that determine the bulk properties of the material. Perhaps, the most important advantage of DEM with respect to the previously mentioned methods is the ability to investigate the physical phenomena occurring at the micro level and its relationship with the macroscopic behaviour. It gives an advantage to DEM over other methods in modelling problems of practical nature because the expected behaviour of a system can be obtained numerically, simply by choosing appropriate microscopic parameters.

The influence of the particle level interaction in the DEM on macroscopic characteristics of wave propagation has been studied by many authors, for instance, Sadd et al. in [8], studied the effects of contact laws on wave attenuation and dispersion behaviour of granular material, whereas in [9] Sadd et al. mainly focused on studying the influence of material microstructure on wave propagation behaviour. Mouraille and Luding [10] investigated dispersion and frequency dependence of wave propagation properties of a regular granular media by exploiting micro–macro transition [15] between particle level interactions and global behaviour. In [16], O’Donovan and O’Sullivan presented a detailed study of the wave velocities and inter-particle contact stiffness using an ideal and relatively simple hexagonal assembly of uniform sized particles. O’Donovan et al. compared experimental results on a model cubical cell of soil with DEM and continuum analysis in [17] and hence was limited only for particular material properties.

In the above mentioned studies, wave propagation in cohesionless granular media has been considered. Propagation of seismic waves in a cohesive material (an intact rock) has been simulated by Toomey and Bean [18] using a regular hexagonal configuration of circular discs. The results have been compared with a high-order finite difference solution of the wave equation. Propagation of elastic waves in rocks and material dynamic properties have been investigated using discrete element particle models by Resende et al. [19]. As a matter of fact, modelling of wave propagation is an inherent part of all discrete element simulations of any problem involving dynamic loading, such as blasting [20], rock cutting [21,22], or rock fragmentation [23].

Micro–macro relationships are essential in material modelling with the DEM. A standard procedure consists in the calibration of the microscopic (contact) model simulating a laboratory quasi-static strength test, such as unconfined compression, shear, Brazilian, or triaxial compression test and apply the calibrated model to simulation of the investigated problem, cf. [21]. It is not always possible to verify if the effective macroscopic properties are represented properly in the discrete element model. The wave propagation problem gives such a possibility. Propagation of ultrasound waves is commonly used as a measurement technique to determine elastic properties. We will exploit the analogous possibility in the numerical analysis to verify if the micro–macro relationships for elastic constants obtained in the simulation of wave propagation correspond to the relationships determined by other methods.

This work presents 2D discrete element modelling of elastic wave propagation in a rock-type cohesive material. Performance of an irregular disc configuration in the simulation of longitudinal and shear elastic waves will be investigated for different values of the shear to normal contact stiffness ratio. Two separate cases will be investigated for the longitudinal mode—propagation in a bulk solid and propagation under uniaxial stress conditions e.g., in a bar. Numerical results will be compared to theoretical predictions for equivalent macroscopic elastic properties determined using the micro–macro relationships obtained by simulation of the uniaxial compression test.

The outline of the paper is as follows. Basic notions of elastic wave propagation are given in Section 2. The formulation of the discrete element method is briefly described in Section 3. Micro–macro constitutive relationships in the DEM have been introduced in Section 4. The micro–macro relationships for elastic properties, Young’s modulus, shear modulus, and Poisson’s ratio, have been determined theoretically and numerically by simulation of the uniaxial compression tests in Section 5. Finally, the results of DEM simulation of the elastic wave propagation have been presented in Section 6. The macroscopic elastic properties in the discrete model have been evaluated based on the longitudinal and shear wave velocities. The corresponding micro–macro relationships have been compared with those obtained by the simulation of the uniaxial compression test (UCT).

## 2. Basic Notions of Elastic Wave Propagation

An elastic wave in a solid body can propagate in two modes, in the form of the longitudinal (also called compression) and transverse (also called shear) waves. In the longitudinal wave, the motion of the material points is in the direction of propagation whereas, in the shear wave, the motion of the material points is in a plane perpendicular to the direction of propagation. Wave propagation velocity, being one of the main parameters characterizing waves, in solid media depends on elastic constants viz. Young’s modulus *E*, Poisson’s ratio ν, and shear modulus *G*, with the following relationships [24]:

(1) $$c_{l}^{bulk}=\sqrt{\frac{E(1-\nu )}{\rho (1+\nu )(1-2\nu )}}\;,$$

for longitudinal wave velocity, $c_{l}^{bulk}$ and

(2) $$c_{s}=\sqrt{\frac{G}{\rho }}=\sqrt{\frac{E}{2\rho (1+\nu )}}\;,$$

for the shear wave velocity, $c_{s}$ with ρ denoting the bulk density. Knowing the wave velocities, the Poisson’s ratio and the Young’s modulus can be deduced by combining Equation (1) with Equation (2) in the following form:

(3) $$\nu =\frac{2-{\left(c_{l}^{bulk}/c_{s}\right)}^{2}}{2\left[1-{\left(c_{l}^{bulk}/c_{s}\right)}^{2}\right]}\;,$$

(4) $$E=\frac{\rho {\left(c_{l}^{bulk}\right)}^{2}\left(1+\nu \right)\left(1-2\nu \right)}{1-\nu }\;,$$

and from the Equation (2), the shear modulus is obtained as:

(5) $$G=\rho c_{s}^{2}\;.$$

The effective macroscopic elastic properties will be determined from the simulations of the bulk longitudinal and shear wave propagation using Equations (3)–(5).

Alternatively, the elastic properties can be determined combining the simulation of the shear wave and the simulation of the longitudinal wave propagation under the uniaxial stress conditions (like in a bar). The relationship for shear wave velocity remains the same as given by Equation (2), and the longitudinal bar wave velocity $c_{l}^{bar}$ is given by [25]:

(6) $$c_{l}^{bar}=\sqrt{\frac{E}{\rho }}\;,$$

The Young’s modulus can be obtained directly from Equation (6):

(7) $$E=\rho {\left(c_{l}^{bar}\right)}^{2}\;,$$

and combining relationships (6) and (2) the Poisson’s ratio can be obtained as:

(8) $$\nu =0.5{\left(\frac{c_{l}^{bar}}{c_{s}}\right)}^{2}-1\;.$$

The shear modulus remains the same as defined by Equation (5).

## 3. Formulation of the Discrete Element Method

Numerical simulations have been performed in this work using the discrete element code DEMPack [26], which has been validated in various applications [21,22,27,28,29]. The underlying formulation of discrete element method implementation following main assumptions of Cundall and Strack [30] is presented below.

### 3.1. Equations of Motion

**Figure 1:**
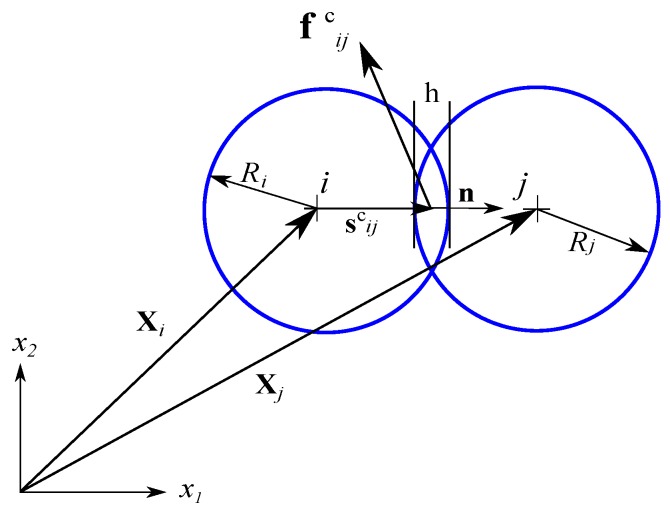
Definition of the inter-particle interaction.

The DEM considers the dynamics of a particulate system. In this work, 2D DEM models employing initially bonded cylindrical particles (discs) have been used. The translational and rotational motion of the discrete elements is described by means of the Newton–Euler equations of rigid body dynamics. For the *i*-th element,

(9) $$m_{i}{\ddot{u}}_{i}=F_{i}\;,$$

(10) $$J_{i}{\dot{\omega }}_{i}=T_{i}\;,$$

where $u_{i}$ is the element centroid displacement in a fixed (inertial) coordinate frame x, $\omega_{i}$—the angular velocity, $m_{i}$—the element mass, $J_{i}$— the moment of inertia. Vectors $F_{i}$ and $T_{i}$ are respectively composed of the forces and moments due to the external load $F_{i}^{\;\mathrm{ext}}$, due to the contact interaction with adjacent particles, $f_{ij}^{\;c}$ and $t_{ij}^{\;c}$, and due to the external damping, $F_{i}^{\;\mathrm{damp}}$ and $T_{i}^{\;\mathrm{damp}}$:

(11) $$F_{i}=F_{i}^{\;\mathrm{ext}}+\sum_{j=1}^{n_{i}^{c}}f_{ij}^{c}+F_{i}^{\;\mathrm{damp}}\;,$$

(12) $$T_{i}=\sum_{j=1}^{n_{i}^{c}}t_{i\;j}^{c}+\sum_{j=1}^{n_{i}^{c}}s_{ij}^{c}\times f_{ij}^{c}+T_{i}^{\;\mathrm{damp}}\;,$$

where $n_{i}^{\;c}$ is the number of elements in contact with the *i*-th discrete element, and $s_{ij}^{\;c}$ is the vector connecting the centre of mass of the *i*-th element with the contact point with the *j*-th element (Figure 1). In the present work, only the force-type contact interaction will be considered, resulting in zero values for the interaction moments $t_{ij}^{\;c}$ of Equation (12).

The damping terms $F_{i}^{\mathrm{damp}}$ and $T_{i}^{\mathrm{damp}}$ in Equations (11) and (12) in the present work are of non-viscous type and are given by:

(13) $$F_{i}^{\mathrm{damp}}=-\alpha^{t}∥F_{i}^{\mathrm{ext}}+\sum_{j=1}^{n_{i}^{c}}f_{ij}^{c}∥\frac{{\dot{u}}_{i}}{∥{\dot{u}}_{i}∥}$$

(14) $$T_{i}^{\mathrm{damp}}=-\alpha^{r}∥\sum_{j=1}^{n_{i}^{c}}s_{ij}^{c}\times f_{ij}^{c}∥\frac{\omega_{i}}{∥\omega_{i}∥}$$

where $\alpha^{t}$ and $\alpha^{r}$, are respective damping factors for translational and rotational motion.

### 3.2. Time Integration Scheme

A second order explicit central difference scheme [31] is employed to integrate Equations (9) and (10). For translation motion, the time integration operator at the *n*-th time step is given as:

(15) $${\ddot{u}}_{i}^{n}=\frac{F_{i}^{n}}{m_{i}},$$

(16) $${\dot{u}}_{i}^{n+1/2}={\dot{u}}_{i}^{n-1/2}+{\ddot{u}}_{i}^{n}\Delta t,$$

(17) $$u_{i}^{n+1}=u_{i}^{n}+{\dot{u}}_{i}^{n+1/2}\Delta t.$$

Similarly, the time integration scheme for the rotational motion can be formulated as:

(18) $${\dot{\omega }}_{i}^{n}=\frac{T_{i}^{n}}{m_{i}},$$

(19) $$\omega_{i}^{n+1/2}=\omega_{i}^{n-1/2}+{\dot{\omega }}_{i}^{n}\Delta t.$$

If required, the rotational configuration can be determined, however, for the disc elements used in this work, the evaluation of rotational configuration is not essential.

The explicit time integration scheme used in DEM imposes a limitation on the time step due to the conditional numerical stability. The time step Δt must not be larger than the critical time step $\Delta t_{cr}$,

(20) $$\Delta t\leq \Delta t_{cr}\;,$$

which is determined by the highest natural frequency of the system, $\xi_{\max}$ as,

(21) $$\Delta t_{cr}=\frac{2}{\xi_{max}}\;.$$

The highest frequency $\xi_{\max}$ can be evaluated by solving the eigenvalue problem defined for the entire system of connected particles, however, this would be computationally expensive. Analogously to the standard simplification proposed for the explicit FEM [32], the maximum frequency of the full system in the DEM can be estimated by natural frequencies of subsets of connected particles surrounding each particle, cf. [33],

(22) $$\xi_{\max}\leq \max\;\xi_{\max}^{\left(i\right)}$$

where $\xi_{\max}^{\left(i\right)}$ is the maximum natural frequency of the system of connected particles surrounding the *i*-th particle. The problem of the critical time evaluation can be simplified further by considering equivalent single degree mass–spring systems with the natural frequency

(23) $$\xi^{\left(i\right)}=\sqrt{\frac{k_{\mathrm{eff}}^{\left(i\right)}}{m_{i}}}$$

where $k_{\mathrm{eff}}^{\left(i\right)}$ is the effective stiffness governing the motion of the *i*-th particle. Hence, the limit on the time step can be given by

(24) $$\Delta t\leq \min\;2\sqrt{\frac{m_{i}}{k_{\mathrm{eff}}^{\left(i\right)}}}$$

The effective stiffness $k_{\mathrm{eff}}^{\left(i\right)}$ depends on the normal and tangential contact stiffnesses, the number of particles connected to the *i*-th particle as well as contact directions, cf. [33]. In practice, the time step can be estimated approximately taking, cf. [34]

(25) $$\Delta t\leq \alpha \sqrt{\frac{m_{\min}}{k_{\max}}}$$

where $m_{\min}$ is the minimum mass and $k_{\max}$ is the largest normal or tangential contact stiffness and α is the user specified parameter accounting for multiple contacts for each mass. For regular packings of equal particles with the same stiffness for all the contacts the parameter α can be determined analytically [33]. For irregular packing a safe value of the parameter α can be based on the results of numerical simulations [34].

### 3.3. Contact Model

The formulation of the contact model employs the decomposition of the contact force between two elements $f^{c}$ into the normal and tangential components, $f_{n}$ and $f_{t}$, respectively:

(26) $$f^{c}=f_{n}+f_{t}=f_{n}n+f_{t}\;,$$

where n is the unit normal vector at the contact point (Figure 1). The normal and tangential contact forces can be evaluated assuming different models [12,35]. Granular materials are usually modelled assuming cohesionless frictional contact [36], while rock-like materials, as well as various other materials, require cohesive contact models [13]. The present work has been focused on the elastic behaviour of the materials modelled with bonded particles, therefore a cohesive contact model is presented. Nevertheless, the formulation presented is valid for a cohesionless contact model, as well.

An initial bonding between the neighbouring particles is assumed. The bonds are established between the neighbouring particles *i* and *j*, if the gap between the particles *g* satisfies the condition:

(27) $$g\leq g_{\max}^{0}$$

where $g_{\max}^{0}$ is a tolerance in the contact verification and the gap *g* is given by

(28) $$g=∥x_{j}-x_{i}∥-R_{i}-R_{j}$$

In the soft contact approach used here, the impenetrability condition is satisfied approximately and a certain overlap between the contact particles *h* is allowed such that,

(29) h=−g>0.

**Figure 2:**
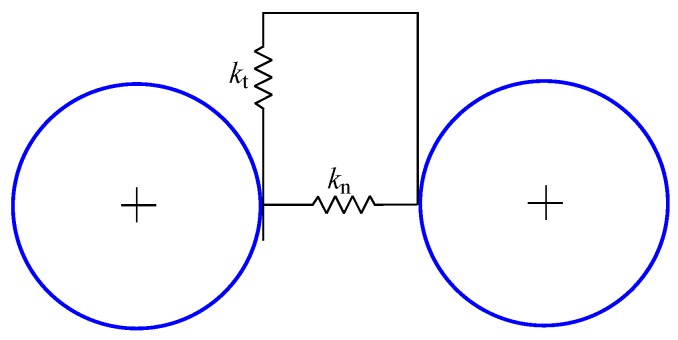
Rheological scheme of the bonded particle interaction model.

The normal and tangential particle interactions are often modelled by linear springs connected in parallel with dashpots, providing an additional mechanism to dissipate contact oscillations. However, in this work the main focus is to investigate the elastic wave propagation, therefore the elastic bonds are assumed here and no additional dissipative mechanisms in the contact model are taken into account (Figure 2). The normal and tangential contact force components are evaluated assuming the linear relationships:

(30) $$f_{n}=k_{n}g\;,$$

(31) $$f_{t}=k_{t}\;u_{t}\;,$$

where $k_{n}$ is the interface stiffness in the normal direction, $k_{t}$—interface stiffness in the tangential direction, and $u_{t}$—the relative displacement at the contact point in the tangential direction. The relative tangential displacement $u_{t}$ must be evaluated incrementally, cf. [33]:

(32) $$u_{t}^{n+1}=u_{t}^{n}+\Delta u_{t}$$

where $u_{t}^{n}$ is the vector of the relative tangential displacement from the previous time step rotated to the present contact plane and $\Delta u_{t}$ is the incremental relative tangential displacement

(33) $$\Delta u_{t}=v_{\mathrm{rt}}^{n+1/2}\Delta t$$

with $v_{t}^{n+1/2}$ being the relative tangential velocity at the contact point determined as

(34) $$v_{\mathrm{rt}}^{n+1/2}={\left(v_{r}^{c}\right)}^{n+1/2}-v_{\mathrm{rn}}^{n+1/2}n$$

where ${\left(v_{r}^{c}\right)}^{n+1/2}$ is the relative velocity at the contact point and $v_{\mathrm{rn}}^{n+1/2}$ its projection on the normal direction:

(35) $$v_{\mathrm{rn}}^{n+1/2}={\left(v_{r}^{c}\right)}^{n+1/2}\cdot n$$

Cohesive bonds are broken instantaneously when the interface strength is exceeded in the tangential direction by the tangential contact force or in the normal direction by the tensile contact force

(36) $$f_{n}\geq \Phi_{n}$$

(37) $$∥f_{t}∥\geq \Phi_{t}$$

where $\Phi_{n}$ in the interface strength in the normal direction, and $\Phi_{t}$ the interface strength in the tangential direction. After decohesion the contact is treated assuming a standard contact model with Coulomb friction. The normal contact force can be compressive only ($F_{n}\leq 0$) and the tangential contact force is limited by $\mu |f_{n}|$

(38) $$∥f_{t}∥\leq \mu |f_{n}|$$

where μ is the Coulomb friction coefficient.

## 4. Micro–Macro Relationships

Obtaining a required macroscopic behaviour by using suitable interparticle contact model and relevant parameters is one of the main difficulties in the DEM. The effect of microscopic parameters in the DEM on macroscopic response have been investigated considerably in literature [37,38,39]. The present work is aimed to investigate the micro–macro relationships between elastic macroscopic properties defined in terms of effective elastic moduli and microscopic DEM parameters. It has been widely indicated in the literature that the macroscopic stiffness parameters depend upon the microscopic parameters such as normal and tangential contact stiffness, $k_{n}$ and $k_{t}$, respectively, particle size $\bar{R}$, porosity, *e*, coordination number [40]. The micro–macro relationships in the DEM can be obtained using various theoretical [41] or numerical [42] methods.

### 4.1. Analytical Micro–Macro Relationships

Effective macroscopic properties of granular materials can be estimated in terms of micromechanical parameters using the kinematic Voigt or static Reuss hypotheses [43]. The kinematic assumption of uniform strain gives the following analytical formulae for the Young’s modulus and Poisson’s ratio for the isotropic packing of equal sized cylindrical discs [41]:

(39) $$E=\frac{4N_{c}R^{2}k_{n}}{V}\cdot \frac{k_{n}+k_{t}}{3k_{n}+k_{t}}\;,$$

(40) $$\nu =\frac{k_{n}-k_{t}}{3k_{n}+k_{t}}\;,$$

where $N_{c}$ is the total number of inter particle contacts in the volume *V*. For the non-uniform particle sized assembly such as used in this work, the arithmetic mean of the radii is generally used. The specimen volume *V* can further be expressed in terms of the particle volume, $V_{p}$ and specimen porosity *e* as follows:

(41) $$V=\frac{N_{p}V_{p}}{1-e}$$

where $N_{p}$ is the number of particles in the specimen and

(42) $$V_{p}=\pi R^{2}t$$

for the disc particle with the thickness, *t*. Thus, by taking into account Equations (41) and (42), the Equation (39) can be written as:

(43) $$E=\frac{2k_{n}n_{c}\left(1-e\right)}{\pi t}\cdot \frac{k_{n}+k_{t}}{3k_{n}+k_{t}}$$

where $n_{c}$ is the coordination number, a parameter defined as an average number of contacts per particle,

(44) $$n_{c}=\frac{2N_{c}}{N_{p}}\;.$$

By rearranging Equation (43), we get the micro–macro relationship for the Young’s modulus in the form,

(45) $$\frac{E\pi t}{2k_{n}n_{c}\left(1-e\right)}=\frac{k_{n}+k_{t}}{3k_{n}+k_{t}}\;.$$

### 4.2. Numerical Micro–Macro Constitutive Relationships

Micro–macro constitutive relationships can be obtained numerically by performing the DEM simulations of laboratory tests, such as the unconfined compression test [44], triaxial test [45], Brazilian test [40], or shear test [46]. Dimensional analysis with the Buckingham π-theorem provides a suitable framework to establish the numerical micro–macro relationships from the DEM simulations in the dimensionless form [44,47].

According to [42] the dimensionless micro–macro constitutive relationships for the Young’s modulus and Poisson’s ratio can be proposed in the following form:

(46) $$\frac{Et}{k_{n}}=\Psi_{E}\left(\frac{k_{t}}{k_{n}},e\right)$$

(47) $$\nu =\Psi_{\nu }\left(\frac{k_{t}}{k_{n}},e\right)$$

where $\Psi_{E}$ and $\Psi_{\nu }$ are certain scaling functions of dimensionless parameters $k_{t}/k_{n}$ and porosity *e*. Following [48] the effect of the specimen porosity and its characteristics can be taken into account using the results of micromechanical considerations expressed by Equations (40) and (45). Thus, the dimensionless (46) and (47) can be rewritten as follows

(48) $$\frac{E\pi t}{2k_{n}n_{c}\left(1-e\right)}={\hat{\Psi }}_{E}\left(\frac{k_{t}}{k_{n}}\right)$$

(49) $$\nu ={\hat{\Psi }}_{\nu }\left(\frac{k_{t}}{k_{n}}\right)$$

where ${\hat{\Psi }}_{E}$ and ${\hat{\Psi }}_{\nu }$ are scaling functions of the dimensionless parameter $k_{t}/k_{n}$.

## 5. Determination of the Micro–Macro Relationships

The dimensionless micro–macro relationships (48) and (49) will be determined numerically by simulation of the uniaxial compression test (UCT) and compared with the analytical ones given by Equations (40) and (45), which have been derived on the basis of Voigt’s kinematic hypothesis.

**Figure 3:**
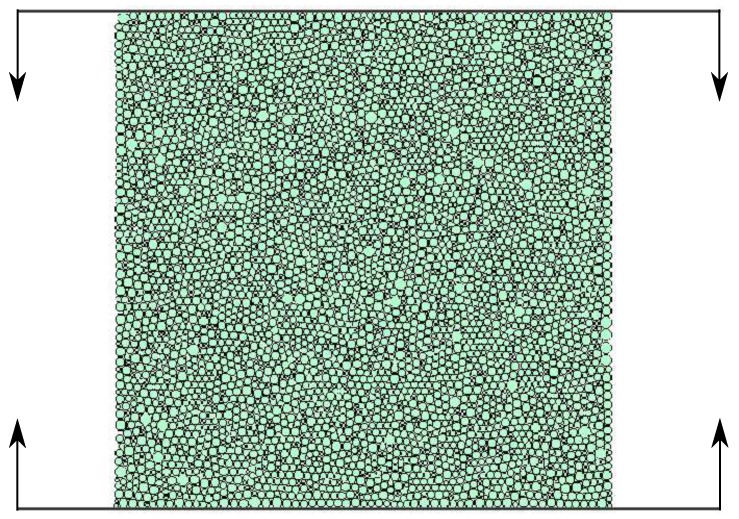
Uniaxial compression test (UCT) of an irregular configuration of nonuniform size particles used for establishing dimensionless constitutive relationships.

Figure 3 shows a square sample 50 mm by 50 mm discretized with 4979 disc shaped elements of nonuniform size with an average radius of 0.370 mm, the maximum and minimum radii being 0.652 mm and 0.218 mm, respectively. The particle packing is characterized by the coordination number, $n_{c}$ = 5.8 and porosity *e* = 0.096. The loading has been introduced by the flat plates moving with a constant velocity 5 mm/s and compressing the specimen through the contact with its top and bottom sides. The microscopic parameters used in these simulations have been the following: particle density ρ=2000 kg/m${}^{3}$, normal contact stiffness $k_{n}=7\times 10^{10}$ N/m. Cohesive bonds strengths $\phi_{n}=\phi_{t}=2.9\times 10^{4}$ N and Coulomb friction coefficient μ=0.83 have been assumed. Additionally, a non-viscous background damping has been used in this example assuming the damping factors $\alpha^{t}=\alpha^{r}=0.2$. Simulations have been conducted for values of the tangential to normal contact stiffness ratio $k_{t}/k_{n}$ ranging between 0.0 to 1.0 in steps of 0.1, obtained by changing the tangential contact stiffness $k_{t}$ for each simulation run.

**Figure 4:**
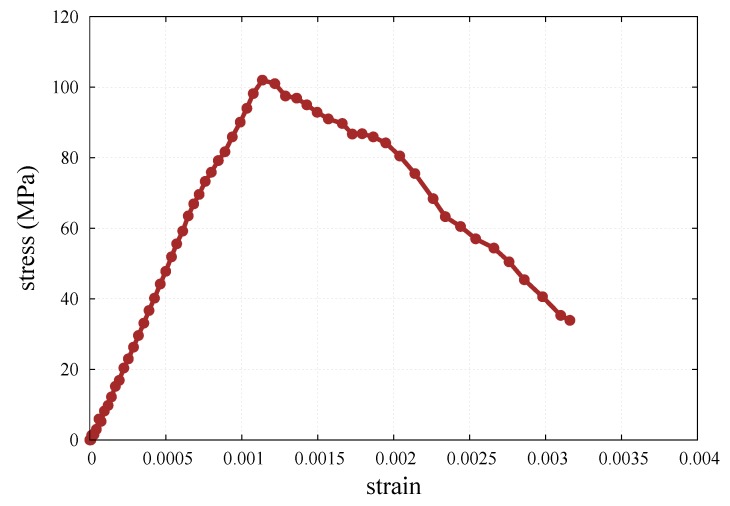
The stress-strain curve for the uniaxial compression numerical test for $k_{t}/k_{n}=0.5$.

Figure 4 presents the macroscopic stress–strain curve for one of the analyses.

The stress–strain curve has been used to determine the macroscopic Young’s modulus *E*:

(50) $$E=\frac{\Delta \sigma_{yy}}{\Delta \varepsilon_{yy}}$$

by taking the stress range from 0 to 50% of the maximum stress level in the considered simulation. The macroscopic Poisson’s ratio has been calculated as

(51) $$\nu =-\frac{\Delta \varepsilon_{xx}}{\Delta \varepsilon_{yy}}$$

where the increments of the strain components correspond to the range used in the determination of the Young’s modulus.

The macroscopic stress, σ has been evaluated in this work by using an averaging procedure involving the internal contact forces in the RVE [43]:

(52) $$\sigma =\frac{1}{V_{RVE}}\sum_{c=1}^{Nc}f^{c}⊗L^{c}$$

where $R_{RVE}$ is the volume of RVE which in the present case is assumed to be the volume, *V* of the sample, and $N_{c}$ is the number of contacts in the RVE. $f^{c}$ denotes the contact force vector at the contact *c*, whereas $L^{c}$ is the vector connecting centroids of two contacting particles and known as the branch vector.

The macroscopic strain tensor for the discrete element assembly has been calculated using the procedure proposed in [40]. Averaging is performed over a triangular mesh generated over the centres of the particles forming the specimen. This is a two level averaging procedure. First, a constant strain $\varepsilon^{k}$ in all the triangles are determined using the formula derived from the averaging equation:

(53) $$\varepsilon^{k}=\frac{1}{S_{k}}\int_{S_{k}}\varepsilon dS\;,$$

where $S_{k}$ is the area of an elementary cell. Applying the divergence theorem, the surface integral in Equation (53) can be transformed into the line integral

(54) $$\int_{S_{k}}\varepsilon dS=\frac{1}{2}\int_{L_{k}}\left(u⊗n+n⊗u\right)dL\;,$$

where $L_{k}$ is the closed boundary of the triangular element, *u*—the displacement field and *n*—the unit normal vector outward to the element. The line integral in Equation (54) is evaluated in terms of nodal displacements and geometric parameters characterizing the triangular element. Having determined the strain $\varepsilon_{ij}^{k}$ in each element the average strain tensor in the whole specimen is obtained by the weighted averaging

(55) $$\bar{\varepsilon }=\frac{1}{S}\sum_{k}S_{k}\varepsilon^{k}\;.$$

**Figure 5:**
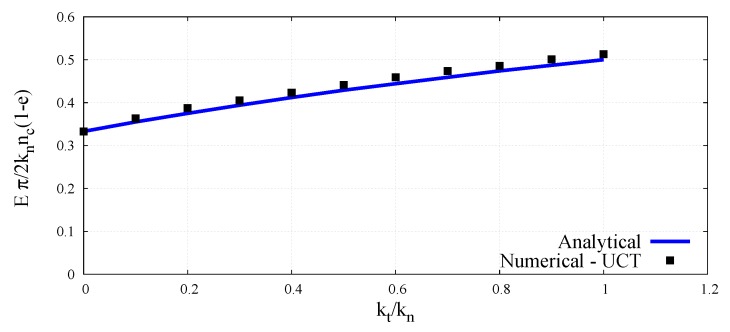
Dimensionless micro–macro relationship for the Young’s modulus as a function of ratio $k_{t}/k_{n}$.

**Figure 6:**
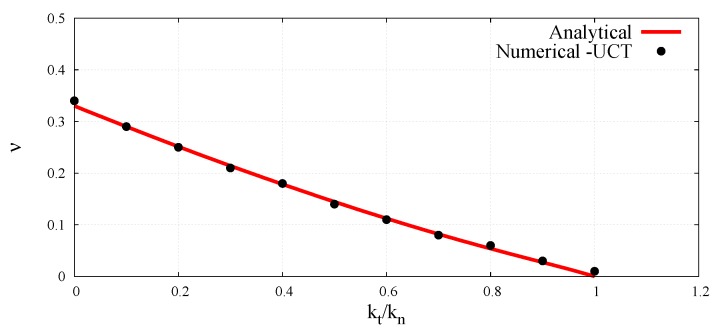
Dimensionless micro–macro relationship for the Poisson’s ratio as a function of ratio $k_{t}/k_{n}$.

With known porosity *e*, normal contact stiffness $k_{n}$, coordination number $n_{c}$, unit particle thickness *t*, and the Young’s modulus determined from Equation (50), the scaling function ${\hat{\Psi }}_{E}$ for the given ratio $k_{t}/k_{n}$ can be calculated according to Equation (48). The values of the scaling function ${\hat{\Psi }}_{\nu }$ defined by Equation (49) are obtained directly from Equation (51). The values of the dimensionless parameter involving the Young’s modulus and Poisson’s ratio for all the simulated cases in the full range of the ratio $k_{t}/k_{n}$ have been plotted in Figure 5 and Figure 6, respectively.

The numerical values have been compared with the analytical predictions according to Equations (40) and (45), which have been derived on the basis of Voigt’s kinematic hypothesis. The close agreement between analytical and numerical values of dimensionless elastic parameters observed here verifies the correctness of dimensionless micro–macro relationships obtained in a static unconfined compression test simulation.

## 6. Simulation of the Wave Propagation

### 6.1. Discrete Element Simulation Setup

**Figure 7:**
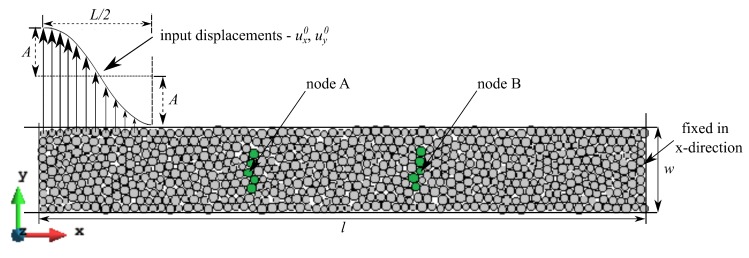
Discrete element sample used in numerical simulations of wave propagation. Disc elements in green color indicate the nodes used to measure the wave velocity in the sample.

The propagation of the plane elastic longitudinal and shear waves has been simulated using a rectangular sample (cf. Figure 7) discretized with 682 bonded disc elements with parameters shown in Table 1. Simulations have been performed for the ratio $k_{t}/k_{n}$ ranging between 0.0 to 1.0 in steps of 0.1. Particles forming the left edge of the sample are free, whereas the right edge of the sample is fixed. Longitudinal waves have been simulated for propagation in a bulk medium and in a bar. For the bulk model, the elements comprising the top and bottom edges are allowed to move only in the direction of wave propagation, whereas free boundary conditions have been applied at the top and bottom edges to obtain uniaxial stress condition for simulating the longitudinal bar wave propagation. The periodic boundary conditions have been imposed for these edges for simulation of the shear wave. The wave impulse has been induced by assigning initial displacements to the particles on the free edge using the following function:

(56) $$u_{i=x,y}^{0}=A\cos\left(\frac{2\pi x}{L}+1\right)$$

where the position of the particles in *x*-direction is bounded within, 0≤x≤L/2 in reference to the left edge of the sample. An amplitude A=0.01 mm and wavelength L=8 mm are assumed, resulting in number of elements per wavelength for the maximum particle radius, $R_{max}$ = 0.145 mm approximately equal to 28 ($L/(2\times R_{max})$). Thus it was ensured that the recommended 20 elements per wavelength are used analogously to the FEM [49]. The initial displacements in the *x* direction have generated the longitudinal wave, while the initial displacements in the *y* direction have been set to induce the shear wave. Wave propagation has been simulated assuming zero damping conditions. Breakage of cohesive bonds has been impeded by setting very high values of bond strength.

### 6.2. Numerical Results

**Figure 8:**
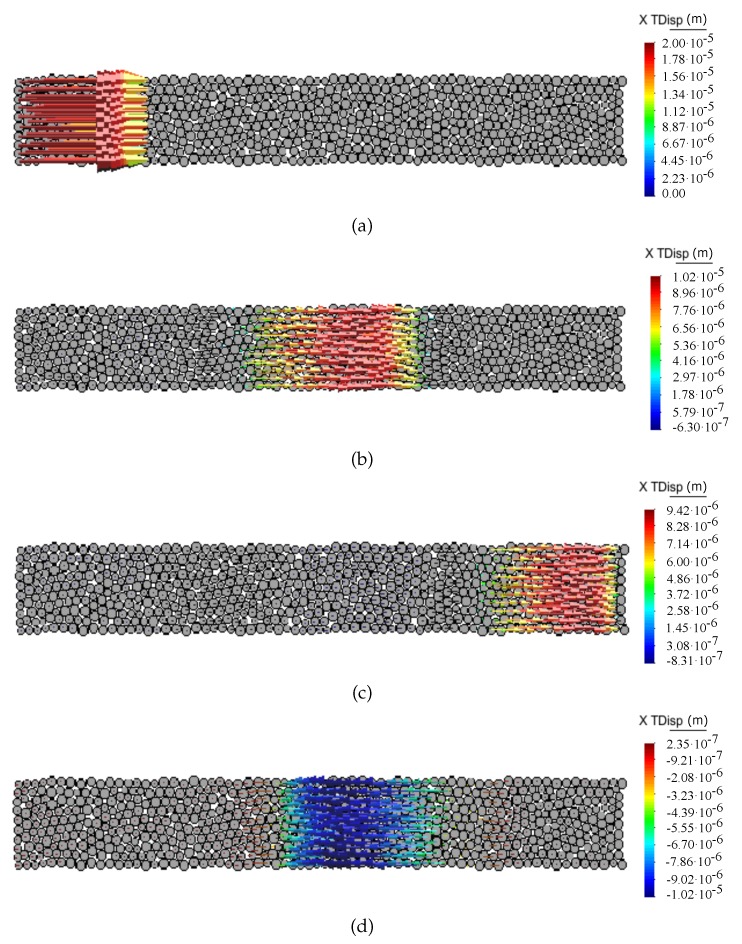
Longitudinal bulk wave propagation through DEM sample. Snapshots of particle displacement vectors captured at time—(**a**) t=0 s, (**b**) $t=3.187\times 10^{-6}$ s, (**c**) $t=5.683\times 10^{-6}$ s, (**d**) $t=8.822\times 10^{-6}$ s.

**Figure 9:**
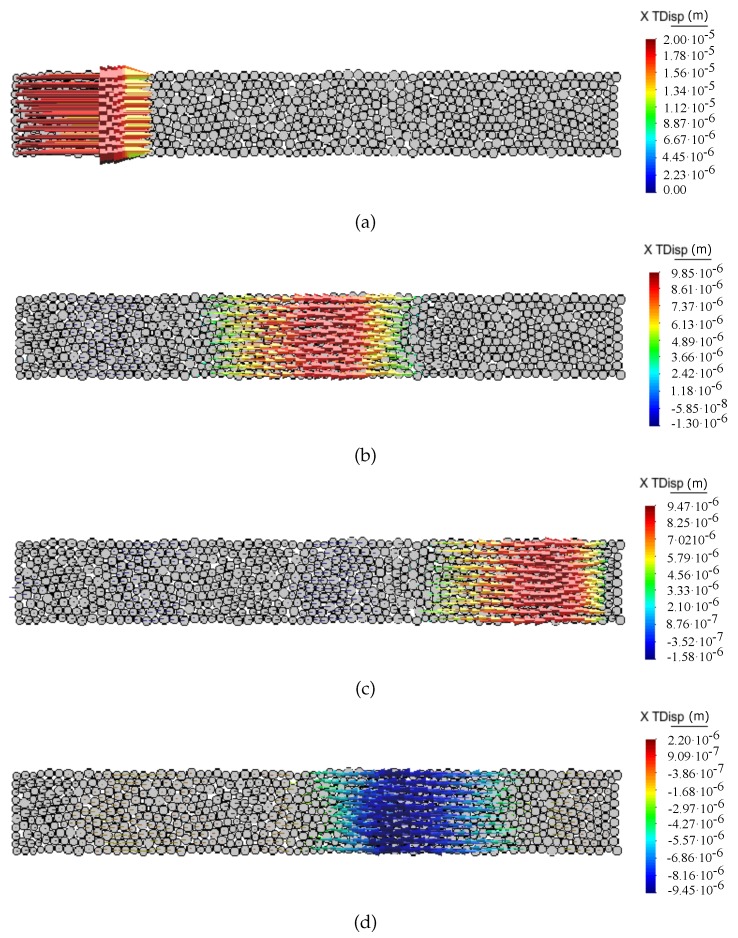
Longitudinal bar wave propagation through DEM sample. Snapshots of particle displacement vectors captured at time—(**a**) t=0 s, (**b**) $t=3.187\times 10^{-6}$ s, (**c**) $t=5.683\times 10^{-6}$ s, (**d**) $t=8.822\times 10^{-6}$ s.

**Figure 10:**
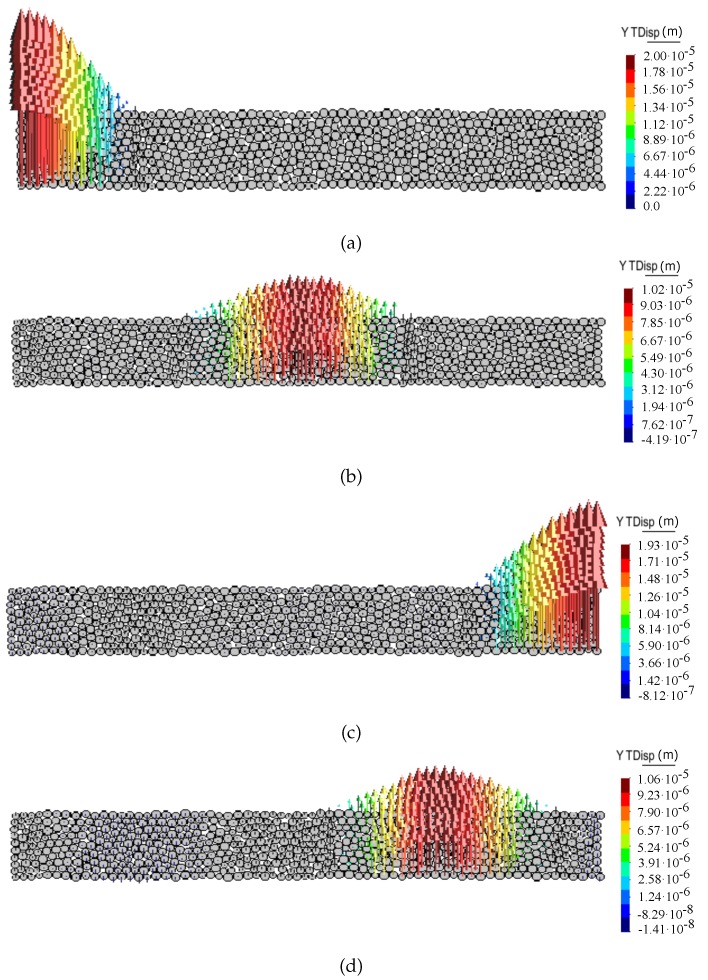
Shear wave propagation through DEM sample. Snapshots of particle displacement vectors captured at time—(**a**) t=0 s, (**b**) $t=5.85\times 10^{-6}$ s, (**c**) $t=1.184\times 10^{-5}$ s, (**d**) $t=1.496\times 10^{-5}$ s.

Figure 8 and Figure 9 show the propagation of the longitudinal wave pulse in a bulk solid and in a bar, respectively, in terms of particle displacement vectors at different time steps for the ratio $k_{t}/k_{n}$ = 0. The shear wave pulse propagation in the discrete sample for the ratio $k_{t}/k_{n}$ = 0 in terms of particle displacement vectors at different time steps is shown in Figure 10.

**Figure 11:**
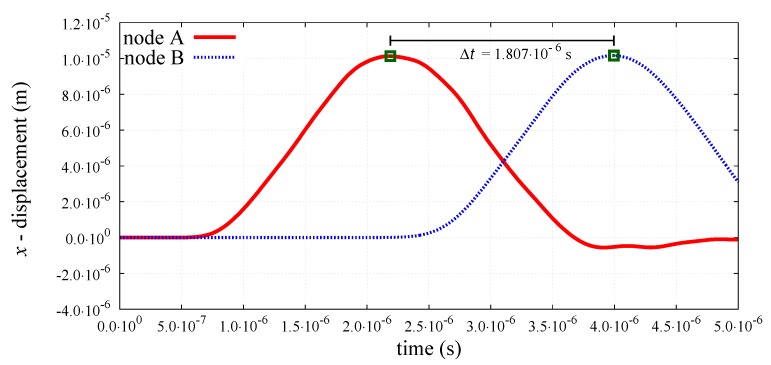
Evolution of *x*-displacement with time for nodes A and B cf. Figure 7 at longitudinal bulk wave propagation.

**Figure 12:**
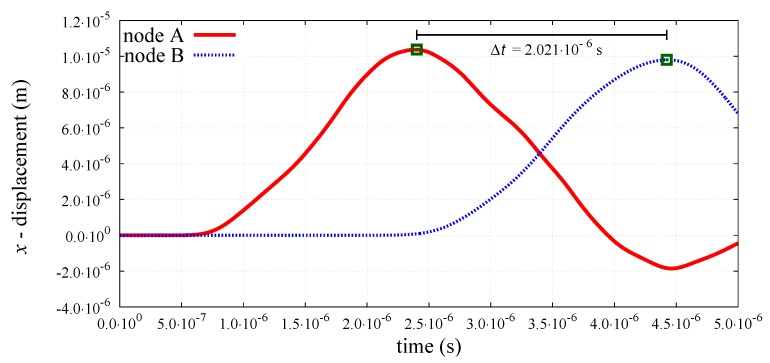
Evolution of *x*-displacement with time for nodes A and B cf. Figure 7 at longitudinal bar wave propagation.

**Figure 13:**
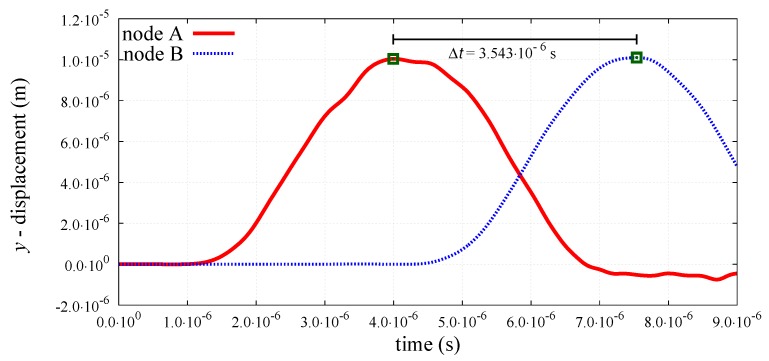
Evolution of *y*-displacement with time for nodes A and B (cf. Figure 7) at shear wave propagation.

**Figure 14:**
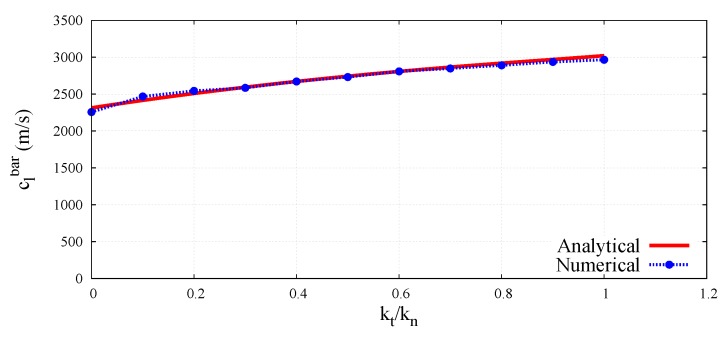
Comparison of longitudinal bar wave velocity, $c_{l}^{bar}$ in DEM sample with analytical results as a function of ratio $k_{t}/k_{n}$.

**Figure 15:**
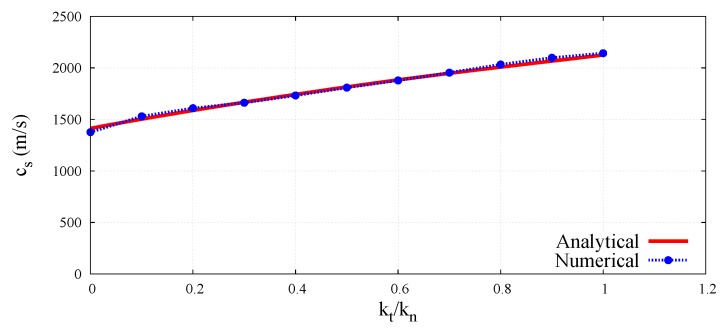
Comparison of shear wave velocity in bulk solid, $c_{s}$ in DEM sample with analytical results as a function of ratio $k_{t}/k_{n}$.

**Figure 16:**
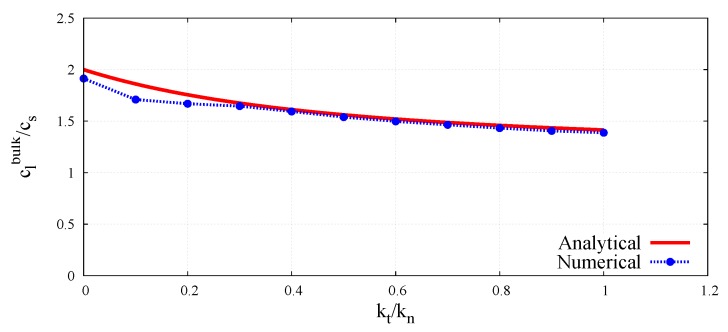
Comparison of longitudinal bulk to shear wave velocity ratio, $c_{l}^{bulk}/c_{s}$ in DEM sample with analytical results as a function of ratio $k_{t}/k_{n}$.

**Figure 17:**
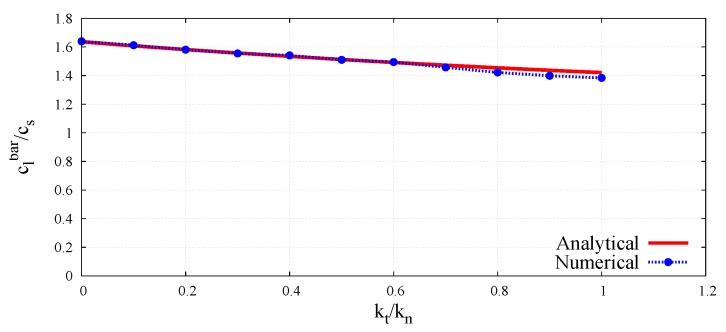
Comparison of longitudinal bar to shear wave velocity ratio, $c_{l}^{bar}/c_{s}$ in DEM sample with analytical results as a function of ratio $k_{t}/k_{n}$.

The peak-to-peak method has been used to evaluate numerical wave velocities from the evolution of particle displacements with respect to time. Displacement time graphs for selected particle pairs for the longitudinal bulk and bar wave have been plotted in Figure 11 and Figure 12, respectively. Displacement time graph for selected particle pairs for the shear wave has been plotted in Figure 13. The time Δt taken by the wave to travel between chosen nodes has been shown in these plots. An average of velocities for five pairs of discrete elements (highlighted with green color in Figure 7) has been used as the velocity for a particular $k_{t}/k_{n}$ ratio. From the DEM simulations results shown in Figure 8, Figure 9, Figure 10, Figure 11, Figure 12 and Figure 13, it can be observed that the longitudinal bulk wave pulse travels with the fastest velocity and the shear wave pulse propagates with the lowest velocity amongst all the three cases. This verifies that in fact by using the DEM the expected wave propagation behaviour can be reproduced. The computational cost (CPU time) for simulating wave propagation cases investigated is quite small. For instance, the CPU time required to simulate the propagation of shear wave impulse through the length of the discrete sample shown in Figure 7, is equal to 7 s with $k_{t}/k_{n}$= 0.0, critical time step $\Delta t_{cr}$ = 1.941 ×$10^{-8}$ s and required 503 time steps. Dependence of the wave velocities on the stiffness ratio $k_{t}/k_{n}$ has been shown in Figure 14 for the longitudinal bar wave and in Figure 15 for the shear wave. Numerical results presented in Figure 14 and Figure 15 have been compared with analytical ones. Analytical wave velocities have been determined as functions of the ratio $k_{t}/k_{n}$ using Equations (2) and (6) with macroscopic Young’s modulus *E* and Poisson’s ratio ν of the discrete sample deduced from Equations (39) and (40), respectively for a given stiffness ratio, $k_{t}/k_{n}$ with no. of contacts, $N_{c}$ = 1852 and average coordination number, $n_{c}=5.43$ for the DEM sample shown in Figure 7. A very good agreement between numerical results and theoretical predictions can be observed in Figure 14 and Figure 15. Similarly, in Figure 16 where longitudinal bulk to shear wave velocity ratio, $c_{l}^{bulk}/c_{s}$ as a function of the ratio $k_{t}/k_{n}$ has been plotted, overall numerical and analytical results agree well, except a few minor deviations at lower values of ratio $k_{t}/k_{n}$. The ratio of longitudinal bar to shear wave velocity, $c_{l}^{bar}/c_{s}$ as a function of the ratio $k_{t}/k_{n}$ has been plotted in Figure 17, where a good agreement between numerical and analytical results can be observed as well.

**Figure 18:**
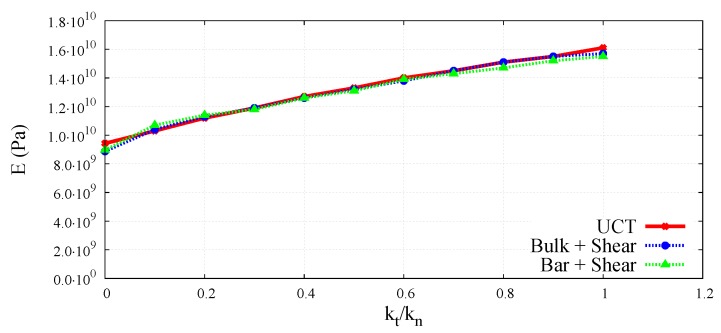
Comparison of Young’s modulus obtained using longitudinal bulk wave velocity with shear wave velocity and longitudinal bar wave velocity with shear wave velocity in DEM sample, with UCT numerical results as a function of ratio $k_{t}/k_{n}$.

**Figure 19:**
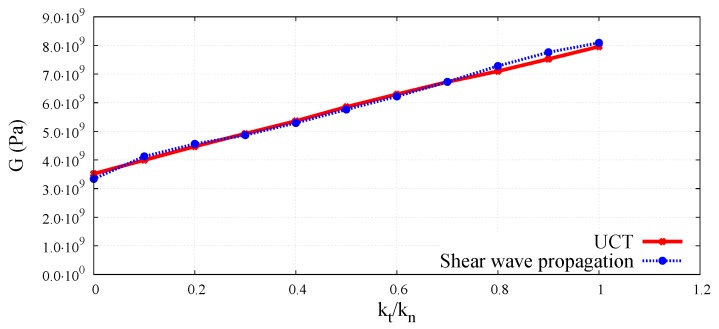
Comparison of shear modulus obtained using shear wave velocity in DEM sample with UCT numerical results as a function of ratio $k_{t}/k_{n}$.

The wave velocities obtained numerically for different $k_{t}/k_{n}$ ratios have been used to evaluate macroscopic elastic parameters for the DEM sample. Equations (3) and (4) have been used to determine effective macroscopic Young’s modulus and Poisson’s ratio from the simulations of the longitudinal bulk wave propagation. Similarly, macroscopic Young’s modulus and Poisson’s ratio for longitudinal wave propagation in a bar have been analysed using Equations (7) and (8). Shear modulus for the transverse wave propagation has been determined from Equation (5). The elastic moduli determined by DEM simulations of the elastic wave propagation have been plotted as functions of the $k_{t}/k_{n}$ ratio in Figure 18 and Figure 19 in comparison with the results obtained by simulations of the uniaxial compression test. The Young’s modulus and Poisson’s ratio corresponding to the simulation of compression test have been evaluated using the dimensionless scale functions given in Figure 5 and Figure 6, respectively, and these parameters have been used subsequently in the relationship, G=E/2(1+ν) for the evaluation of shear modulus.

**Figure 20:**
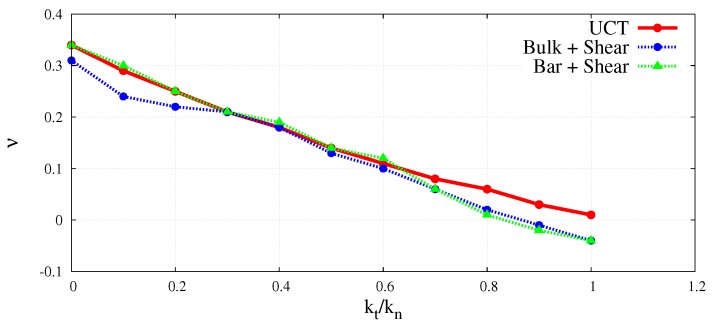
Comparison of Poisson’s ratio obtained using longitudinal bulk wave velocity with shear wave velocity and longitudinal bar wave velocity with shear wave velocity in DEM sample, with UCT numerical results as a function of ratio $k_{t}/k_{n}$.

A very good agreement between the Young’s moduli obtained through dynamic and quasistatic numerical simulations in the full range of ratio $k_{t}/k_{n}$ between 0.0 and 1.0 can be seen in Figure 18. Similarly, a close concurrence between Shear moduli obtained from the dynamic and quasistatic numerical simulations for the entire range of the ratio $k_{t}/k_{n}$ can be noticed in Figure 19. A certain deviation of dynamic results from the quasistatic data can be observed in Figure 20 for some values of the Poisson’s ratio. This can be explained by analysing the form of Equation (3) for the wave propagation in a bulk solid and Equation (8) for the wave propagation in a bar. It can be deduced that the numerical evaluation of Poisson’s ratio is highly sensitive to the numerical evaluation of the wave velocity ratios $c_{l}^{bulk}/c_{s}$ and $c_{l}^{bar}/c_{s}$. Even a small numerical error in the evaluation of wave velocities leads to the square of the error in Poisson’s ratio calculation. For small values of the Poisson’s ratio (close to zero) the numerical error can be of the order of the evaluated parameter value.

## 7. Conclusions

Numerical simulations have confirmed the capability of the discrete element method to represent properly phenomenon of the elastic wave propagation in a solid material both in longitudinal and shear modes. The wave propagation velocities agree well with theoretical predictions. This allows to use wave velocities to determine elastic macroscopic properties of solid media discretized with discrete elements. Comparisons with micro–macro relationships obtained numerically by simulations of the quasistatic compression test show that the values of the Young’s and shear moduli based on the wave velocities agree very well with those determined by simulations of the quasistatic compression test, however, there is a certain difference for the Poisson’s ratio in the range of its low values (lower than 0.1). Calculation of the Poisson’s ratio in this range is very sensitive to inaccuracies in evaluation of wave velocities.

## Figures and Tables

**Table 1:** Parameters of discrete element method (DEM) sample used in numerical studies of wave propagation.

Symbol	Parameter	Value	Units
$N_{p}$	no. of particles	682	-
$R_{max.}$	max. radius	0.145	mm
$R_{min.}$	min. radius	0.1	mm
*l*	sample length	16.54	mm
*w*	sample width	2.3	mm
*e*	porosity	0.11	-
$\rho_{p}$	particle density	2000.0	kg/m${}^{3}$
$\rho_{avg.}$	sample average density	1784.26	kg/m${}^{3}$
$k_{n}$	normal contact stiffness	1 × $10^{10}$	N/m
